# Electrospun Magnetic Nanofiber Mats for Magnetic Hyperthermia in Cancer Treatment Applications—Technology, Mechanism, and Materials

**DOI:** 10.3390/polym15081902

**Published:** 2023-04-15

**Authors:** Al Mamun, Lilia Sabantina

**Affiliations:** 1 Junior Research Group “Nanomaterials”, Faculty of Engineering and Mathematics, Bielefeld University of Applied Sciences, 33619 Bielefeld, Germany; 2 Faculty of Clothing Technology and Garment Engineering, HTW-Berlin University of Applied Sciences, 12459 Berlin, Germany

**Keywords:** magnetic hyperthermia, electrospun magnetic nanofiber mats, magnetic nanomaterials, targeted drug delivery, cancer treatment, magnetic nanofibers

## Abstract

The number of cancer patients is rapidly increasing worldwide. Among the leading causes of human death, cancer can be regarded as one of the major threats to humans. Although many new cancer treatment procedures such as chemotherapy, radiotherapy, and surgical methods are nowadays being developed and used for testing purposes, results show limited efficiency and high toxicity, even if they have the potential to damage cancer cells in the process. In contrast, magnetic hyperthermia is a field that originated from the use of magnetic nanomaterials, which, due to their magnetic properties and other characteristics, are used in many clinical trials as one of the solutions for cancer treatment. Magnetic nanomaterials can increase the temperature of nanoparticles located in tumor tissue by applying an alternating magnetic field. A very simple, inexpensive, and environmentally friendly method is the fabrication of various types of functional nanostructures by adding magnetic additives to the spinning solution in the electrospinning process, which can overcome the limitations of this challenging treatment process. Here, we review recently developed electrospun magnetic nanofiber mats and magnetic nanomaterials that support magnetic hyperthermia therapy, targeted drug delivery, diagnostic and therapeutic tools, and techniques for cancer treatment.

## 1. Introduction

**Figure 1:**
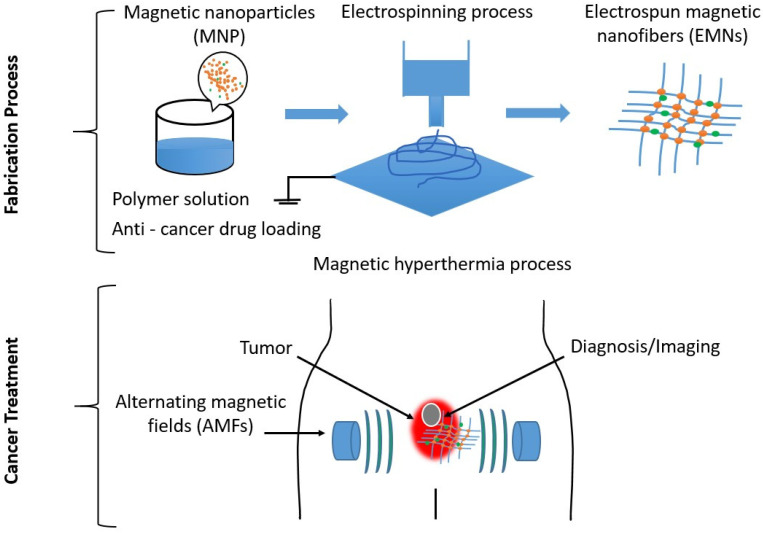
Graphical abstract showing the use of electrospun magnetic nanofiber mats in therapeutic and diagnostic methods for cancer treatment.

According to statistical data from the International Agency for Research on Cancer, the number of cancer deaths worldwide in 2020 was estimated at around 9.96 million, and new cancer cases were estimated at around 19.3 million [1]. The number of global cancer cases is projected to increase to 28.4 million by 2040, representing an increase of nearly 50% in the next 20 years [2], making the disease a great threat to human beings worldwide [3,4]. However, various treatment modalities such as hyperthermia, radiotherapy, chemotherapy, photodynamic therapy, hormone therapy, immunotherapy, stem cell transplantation, surgery, biomarker testing, etc., are currently being developed and tested to address this global issue [5,6,7,8]. Among these, chemotherapy, radiotherapy, and hyperthermia have proved to be successful treatment methods that can kill cancer cells [8,9,10]. Nevertheless, toxicity and side effects are very high in chemotherapy and radiotherapy methods [11,12]. For this reason, hyperthermia is the most attractive and effective recent treatment method that does not have toxicity to healthy tissues [13,14,15]. Specific hyperthermia therapy comprises three treatment modalities: whole-body hyperthermia, regional hyperthermia, and local hyperthermia. If cancer cells are detected in the initial stage, local hyperthermia is applied; if the affected area is larger than the tumor, regional hyperthermia is applied to a complete tissue or organ; if cancer is detected in the final stage, the affected cells are distributed throughout the body, and whole-body hyperthermia should be applied [16]. In the final case, the treatment process can be more complicated and harmful and can sometimes damage healthy tissue [17,18,19]. Therefore, it is very important to detect tumor cells in their early stages. During hyperthermia treatment, heat is generated in the tumor area to kill the affected cells by introducing external substances into the tumor [20]. Affected cancer cells die as soon as the temperature exceeds 42 °C because cancer cells are more sensitive than healthy cells [21,22,23]. Healthy cells, in contrast, can survive at this temperature [24]. Several methods for energy transfer with external devices are used to generate temperature in the target tissue [25]. For this process, laser, ultrasound, induction heating, electromagnetic waves, radio frequency, microwaves, infrared radiation, etc., are usually used to generate heat [26,27,28]. However, the traditional hyperthermia procedure has some limitations and challenges due to insufficient penetration of heat waves into tissues when using lasers, ultrasound, and microwaves, overheating of healthy cells, and severe side effects occurring as a result of combustion. Recently, medical scientists, in collaboration with materials scientists, have developed magnetic nanostructures to generate heat for this purpose using magnetic nanomaterials, especially iron oxide nanoparticles with superparamagnetic behavior, as these materials can generate heat under an alternating magnetic field [29,30,31]. In addition, magnetic hyperthermia is more effective and less harmful to healthy cells, and it can also help overcome the limitations of the traditional hyperthermia process. In contrast, therapeutic and diagnostic materials, such as theranostic devices, have recently become a very interesting research area in the medical field to enable faster and more effective cancer treatment, imaging, and diagnosis [32,33,34]. Figure 1 shows a graphical abstract of the production of electrospun magnetic nanofiber mats, which can also be conditionally loaded with drugs for use in therapeutic and diagnostic procedures for cancer treatment.

**Figure 2:**
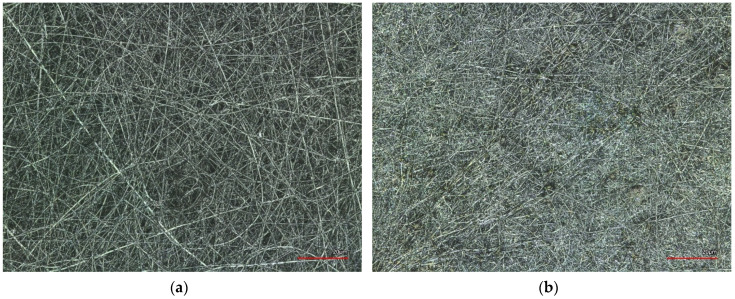
Confocal laser scanning microscope (CLSM) images showing a nanofiber mat with biopolymer lignin (10 wt% PEO and 1 wt% lignin) (**a**) and a nanofiber mat with PAN/magnetite (10 wt% PAN and 5 wt% Fe_3_O_4_ (**b**). Scale bars indicate 20 μm.

Moreover, the most suitable and recently developed materials, especially magnetic nanomaterials, are used for these purposes [35]. Electrospun nanofiber mats with magnetic additives are promising materials for biomedical applications [36,37] such as targeted drug release in tumors and theranostic devices for cancer treatment. They have great potential in this field due to the unique physical and chemical properties of electrospun nanofibers, including their networks of nanoporous and microporous structures, their large specific surface-to-volume ratio, tunable porosity, and flexible surface functionality [38,39,40,41,42,43]. Electrospinning is a very simple, cost-effective, and environmentally friendly process for producing nanofibers from various polymers [44] and even ceramics [45]. In addition, electrospun magnetic nanofiber mats can be produced from bio- or natural polymer solutions with magnetic additives [46]. Figure 2 shows the confocal laser scanning microscope (CLSM) images of a nanofiber mat with biopolymer lignin (10 wt% PEO and 1 wt% lignin) and a nanofiber mat with polyacrylonitrile (PAN)/magnetite (10 wt% PAN and 5 wt% Fe_3_O_4_).

Electrospun magnetic nanofiber mats show special characteristics with regard to materials, size and shape, and physical, chemical, and magnetic properties [47,48]. Therefore, these newly developed electrospun magnetic nanofiber mats are not only a promising material for magnetic hyperthermia in cancer treatment but can also be used for diagnostic purposes such as imaging to detect early cancer stages [49,50].

There have been several studies on the use of magnetic electrospun nanofibers for hyperthermia in cancer therapy, but there are only a few reviews. For instance, Vilas-Boas et al. conducted in vitro and in vivo studies to identify the parameters that affect magnetic hyperthermia [51], while Włodarczyk et al. focused on the challenges associated with the use of nanoparticles in magnetic hyperthermia [52]. Additionally, Govindan et al. explored the use of artificial intelligence in magnetic hyperthermia therapy [53]. However, these reviews mainly focused on hyperthermia therapy without the use of nanofibers. In contrast, the review by Soares and Borges centered on the use of magnetic nanofibers for cancer therapy; however, they mainly discussed the magnetic nanoparticles and polymers used in their production [54]. This particular review, on the other hand, focuses specifically on hyperthermia treatment using magnetic nanofibers and electrospinning technologies. It also discusses the materials used and the mechanism of magnetic hyperthermia for cancer therapy.

This review paper outlines some of the aspects of the current and upcoming advancement in magnetic nanostructures based on electrospun magnetic nanofiber mats and magnetic nanomaterials in cancer theranostics that are relevant to medicine and advanced materials science. In addition, it also provides a brief overview of electrospinning technology, the mechanism of magnetic hyperthermia, electrospun magnetic nanofiber mats used for hyperthermia, challenges and limitations, and the future perspective of cancer theranostics.

## 2. Fabrication Technique for Electrospun Magnetic Nanofiber Mats

Nanofiber and electrospun nanofiber mat fabrication technology is an interesting research area for scientists. Various methods for fabricating nanofiber mats have been described in the literature, such as melt electrospinning, self-bundling, multi-nozzle electrospinning, bubble electrospinning, electro-blowing, cylindrical porous hollow tube electrospinning, and electrospinning. Electrospinning is a very simple, easy, environmentally friendly, inexpensive, and popular method for fabricating nanofibers [55,56]. It can be used to produce very fine fibers or fiber mats with diameters in the nanometer range for both academic and industrial research purposes. Depending on their application, electrospun nanofiber mats can be made from a variety of materials, such as natural or biopolymers, polymer composites or melts, inorganic or inorganic–organic materials, metallic nanoparticles, particulates, carbon nanotubes, and even ceramics [57,58,59].

**Figure 3:**
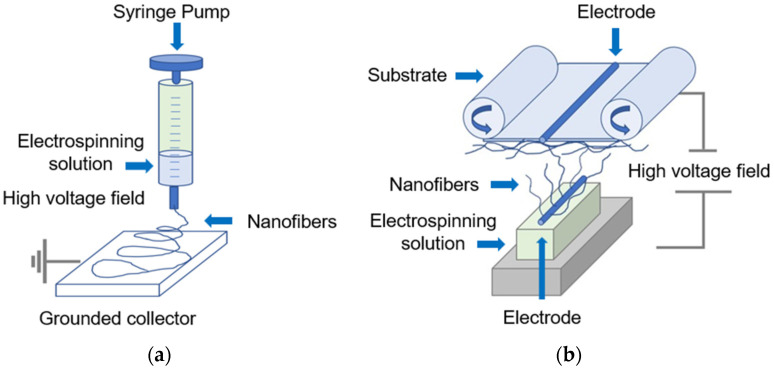
Overview of needle-based (**a**) and needle-free (**b**) electrospinning technologies. Adapted from [60], originally published under a CC-BY license.

In general, there are two types of electrospinning processes: needle-based electrospinning and needle-free electrospinning (see Figure 3).

The standard needle-based electrospinning systems can be set up either vertically or horizontally, while needle-free electrospinning has either rotating or stationary spinnerets depending on the working conditions. The production of electrospun nanofiber mats in both types of electrospinning processes depends on some parameters, including solution, operating and environmental parameters, etc. Needle-based electrospinning requires a high-voltage supply and a dosing device for the spinning solution connected to the spinnerets, an adjustable control system, and a reservoir for the polymer solution (e.g., a small-diameter syringe) to facilitate the production of nanofibers [60,61] (see Figure 3a). Needle-free electrospinning, in contrast, is a self-organized process that enables the electrospinning of nanofibers directly from an open liquid platform (see Figure 3b). Throughout the process, a thin layer of a polymer solution is deposited on the surface of the spinneret and continuously rotated. This rotation creates conical peaks on the surface of the solution. At the same time, the electrical forces create Taylor cones and thus nanofibers. During the preparation of the electrospun solution, the polymers dissolve in a solvent and form a polymeric electrospun solution. Materials can be added to produce modified electrospun nanofiber mats depending on their application [62]. Most of the polymers used to fabricate nanofibers are dissolved in harmful to highly hazardous organic solvents such as dimethylformamide (DMF), dimethyl sulfoxide (DMSO), dimethylacetamide (DMAc), dimethyl sulfone, tetramethyl sulfide, and aqueous solutions of ethylene carbonate and some mineral salts [63,64]. According to the scientific literature, most nanofibers are spun from toxic solvents. Some of them are highly flammable, toxic, harmful to the environment, require special disposal, can harm humans, and have high health and safety requirements [65], and only some papers report the use of the low-toxicity solvent dimethyl sulfoxide (DMSO). Therefore, there is a high demand and interest in “green electrospinning” and low-toxicity solvents, such as dimethyl sulfoxide (DMSO), that are easier to handle and do not cause disposal problems [66,67,68]. Electrospun nanofiber mats can be made from natural or synthetic polymers, including cellulose, gelatin, casein and alginate, chitosan, polysaccharides, collagen, keratin, silk, tubulin, actin, fibrin, poly(ethylene glycol) (PEG), polyacrylonitrile (PAN), poly(lactic acid) (PLA), poly(L-lactic acid) (PLLA), acrylonitrile-butadiene-styrene (ABS), poly(lactic-co-glycolic acid) (PLGA), polyurethane (PU), polyvinyl alcohol (PVA), polycaprolactone (PCL), and polymethyl methacrylate (PMMA). These and many other polymers are known to be suitable for medical applications. In addition, magnetic additives, such as all ferrimagnetic iron oxides, Co, Ni, etc., can be processed into electrospun magnetic nanofiber mats by adding them into a polymer solution for magnetic hyperthermia [69,70,71,72,73,74].

## 3. Magnetic Hyperthermia Process and Materials

### 3.1. Hyperthermia

Hyperthermia is a procedure used in medical science to treat cancer by heating cancer cells, and thus killing them, using various techniques. Depending on the targeted cancer cells, temperatures range from 39 °C to 46 °C [75,76,77,78,79,80,81]. Some essential components to increase the temperature of tumor cells include ultrasound, radiofrequency (in the range from 100 kHz to 150 MHz), microwaves (wavelengths from 433 to 2450 MHz), hot water perfusion (pipes, ceilings), infrared emitters, nanoparticles, magnetic iron oxide nanoparticles, and resistive wire implants in a hyperthermia system [82,83,84,85,86,87,88,89,90,91].

### 3.2. Magnetic Hyperthermia

Research has found that magnetic hyperthermia is a novel procedure that offers a safe, powerful, and simple treatment method to meet these complicated challenges [92,93,94,95,96]. In this procedure, which has recently seen rapid development, magnetic nanomaterials can be used to improve hyperthermia efficiency compared to traditional hyperthermia methods for the erosion of tumors [97,98,99,100,101,102,103,104,105,106]. The most significant aspect of magnetic hyperthermia is that magnetic nanomaterials are distributed over very small areas so that the temperature behavior of healthy cells is not affected [107,108,109]. For this purpose, the highest possible saturation magnetization can be achieved with multifunctional iron oxide-based magnetic nanomaterials used on the specific therapeutic side of cancer cells. These magnetic nanomaterials are capable of generating thermal energy when an external magnetic field is applied. In magnetic hyperthermia, two types of external fields are applied: dynamic/oscillating and static [110,111,112,113,114,115,116]. In an oscillating field, an alternating current source is connected to the field, which fluctuates with frequency. It is referred to as an alternating magnetic field. Therefore, two different methods—switching the external magnetic field and changing the direction of the magnetic field—can be used to release this alternative magnetic field for magnetic hyperthermia [117]. Moreover, these low-frequency alternating magnetic fields (100 kHz–1 MHz) can deeply penetrate the body without causing significant attenuation losses [118,119,120,121]. Alternating magnetic fields with amplitudes of tens of kA/m and frequencies of 100 kHz and 1 MHz are applied in the target area so that the magnetic energy of the magnetic nanomaterials is directed toward the applied magnetic field [122,123,124,125,126]. The magnetic energy is transformed into thermal energy under different conditions, which can increase the temperature of cancerous tissue and consequently cause apoptosis or necrosis in the tumor [127,128,129,130,131]. It is known that iron-based magnetic nanomaterials can destroy the affected cells without being toxic, and the aggregated nanomaterials are excreted from the body after several weeks [132,133,134,135,136]. The main mechanisms of heat generation under an alternative magnetic field based on the properties of magnetic nanomaterials are as follows: i. hysteresis power loss of the magnetic nanoparticles, ii. Néel relaxation, and iii. frictional losses due to Brownian rotation in the magnetic particles. Applied gradually, these mechanisms lead to a saturation of thermal energy [137,138,139,140,141,142,143].

### 3.3. Magnetic Hyperthermia Involving Magnetic Nanomaterials

**Figure 4:**
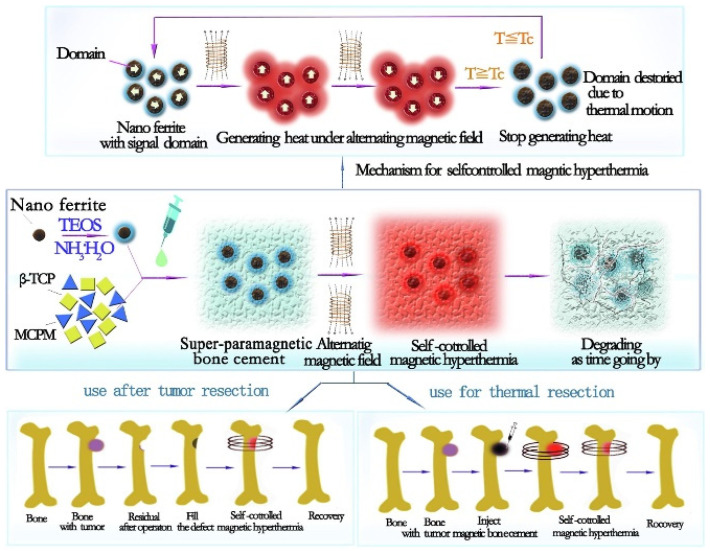
Theoretical schematic diagram showing the design of a magnetic nanomaterial for magnetic hyperthermia. Reproduced with permission from [167].

Due to the magnetic and superparamagnetic properties of nanomagnetic materials, they are becoming increasingly attractive nano-devices for medical science aiming to improve diagnostic precision and the treatment of diseases, especially cancer [144,145,146,147,148,149,150,151,152,153,154,155,156,157]. The main sources of magnetic nanoparticles are pure metals, their oxides, or metal alloys. Therefore, due to the high magnetization and oxidation properties of pure metal, as well as their high toxicity, metal oxide nanomaterials (such as magnetite, maghemite, and other ferrites, including Co, Mn, Ni, Zn, and others) are preferable for biomedical applications [158,159,160,161]. Their superparamagnetic properties, biocompatibility, and chemical stability are excellent characteristics for this purpose. In addition, magnetic nanomaterials respond to the alternative magnetic field by generating thermal energy, which is effective in magnetic hyperthermia. With a specific nanometer size of less than 20 nm, Fe_3_O_4_ is the most attractive magnetic nanomaterial due to its properties, which include superparamagnetic, high saturation magnetization, soft magnetic behavior, suitable particle shape and size, easy synthesis, and low density [162,163]. In addition, thermal efficiency depends on the main function of intermolecular interactions, dipolar interaction between particles, particle size and geometry, saturation magnetization, relaxation time (Néel and Brown), magnetic anisotropy, and superparamagnetic properties [164,165,166]. The generated heat is mainly due to the energy lost in overcoming the rotational energy barrier of the alternating magnetic field for a superparamagnetic nanomaterial smaller than the single-domain region (see Figure 4). 

Consequently, such magnetic nanomaterials, possibly combined with polymeric materials, can be used in cancer treatment as a therapeutic device platform for drug release, as imaging probes for cancer diagnostics, and for magnetic hyperthermia [168,169,170,171,172].

### 3.4. Mechanism of Thermal Energy Generation Using Magnetic Nanomaterials

**Figure 5:**
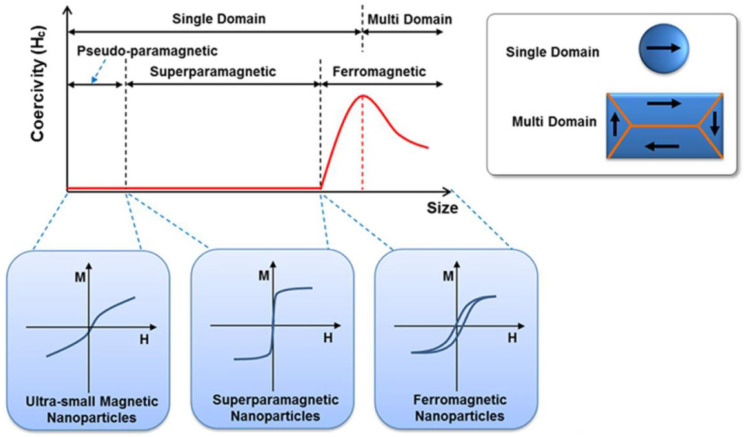
Overview showing the mechanism of thermal energy generation using magnetic nanomaterials. Visualization of typical hysteresis loops in a ferromagnetic/ferrimagnetic nanomaterial and a superparamagnetic nanomaterial and the dependence of the coercivity on the particle size. Adapted from [182], originally published under a CC-BY license.

Normally, magnetic hysteresis losses are observed in magnetic nanomaterials when an external magnetic field is applied [173,174]. These magnetic losses largely depend on the magnetic features of the magnetic nanomaterials based on their size [175]. For example, a multi-domain state is evident in bulk materials. However, the thermal energy generated using both single- and multi-domain magnetic nanomaterials is always a function of hysteresis losses, which depends on how fast the magnetization follows the alternating magnetic field (AMF) changes [176,177,178,179,180,181]. Moreover, the amount of magnetic energy transformed into heat during magnetization reversal follows the magnetic loss in the magnetic nanoparticles. Thus, the thermal energy generated using the magnetic nanomaterial is approximately equal to the area of the hysteresis loop formed during one cycle of the magnetic field (see Figure 5) [182,183,184].

In addition, movement is required to overcome the friction between the magnetization easy axis and the atomic lattices for the Néel relaxation or between magnetic nanomaterials and their surroundings for Brownian relaxation, leading to the loss of electromagnetic energy and the production of thermal energy [185,186,187].

### 3.5. Magnetic Hyperthermia with Electrospun Magnetic Nanofiber Mats

The potential biomedical applications of magnetic nanostructures can be enhanced by adding magnetic nanomaterials to composite polymers [188,189]. The large surface area of the nanomaterials, the irregular composition, and the shape of the magnetic nanomaterials lead to an imbalance in dipolar attraction and a strong interaction between the particles [190,191]. The temperature, concentration of nanomaterials, surface charge of nanoparticles, dielectric constant, ionic strength of the medium, presence of surfactants, magnetic attraction force, high surface energy, and van der Waals forces are the basic parameters that lead to the agglomeration of magnetic nanomaterials to the polymer matrix [192,193]. Magnetic particles generate heat when exposed to an external alternating magnetic field by various physical mechanisms such as relaxation loss or hysteresis loss [194,195,196]. All hysteresis losses occur in the hysteresis loop area of superparamagnetic or ferromagnetic/ferrimagnetic nanomaterials [197,198,199,200,201]. As the magnetic nanomaterials are fixed inside the electrospun magnetic nanofiber mats, they generally cannot rotate freely in the applied magnetic field. Typically, magnetic materials can experience heating through any of the four distinct mechanisms when subjected to high-frequency magnetic fields, namely eddy currents and hysteresis loss, as well as Brownian and Néel relaxation [202,203,204]. Zhong et al. have shown, for example, that for fixed magnetic nanomaterials in the polymer matrix, magnetic reversal losses are responsible for heat generation. Since the magnetic nanomaterials are incorporated into the fiber mats and thus fixed, the complete rotation of the nanomaterials (extrinsic relaxation) can be avoided. From the hysteresis curve of the nanomaterials, a coercivity of about 80 Oe and a relative remanence of 0.12 were determined. These values are a clear indication of a dominant ferrimagnetic behavior of the nanomaterials in the fibers, so hysteresis will be the main loss mechanism in magnetization reversal. Due to the size distribution of the magnetic nanomaterials, there will also be a fraction of very small superparamagnetic nanomaterials within the particle ensemble. Their magnetization will be reversed via Néel relaxation [205].

The magnetization dynamics of low-dimensional objects consist of many factors, e.g., the geometric shape, which determines the prevailing competition between demagnetization and exchange energy, as shown in the study by Steblinski et al. [206]. Using electrospinning techniques, magnetic nanoparticles can be embedded into electrospun nanofibers and other polymeric matrices to create defined magnetic and mechanical properties. The metal oxide nanoparticles have a strong tendency to form agglomerations—an effect that, as a consequence, changes the magnetic properties of the composites. The study by Blachowicz et al. investigated metal oxide nanoparticles such as magnetite or nickel ferrite and their embedding into a polymer to avoid oxidation. It also investigated the influence of agglomeration on the magnetic properties of metal oxide nanoparticles with different diameters in non-magnetic matrices [207].

**Figure 6:**
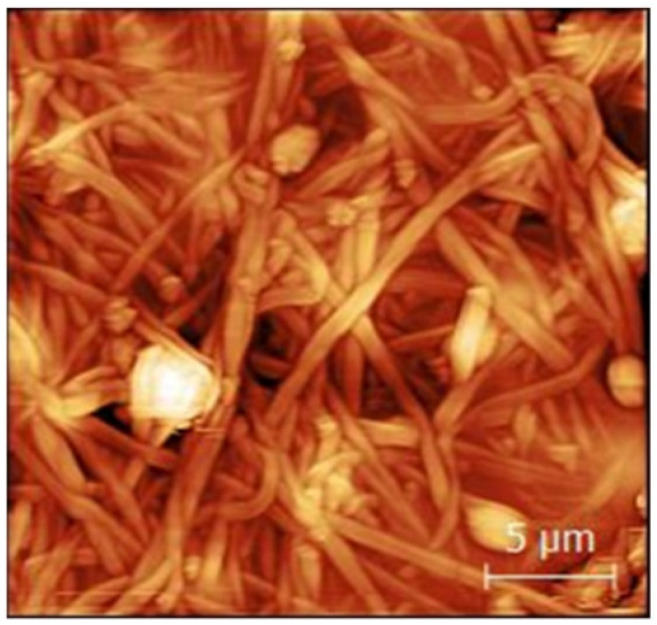
Atomic force microscopic (AFM) image showing a magnetic electrospun PAN nanofiber mat with 20 wt% Fe_3_O_4_. The scale bar indicates 5 μm. Adapted from [208], originally published under a CC-BY license.

Figure 6 shows the atomic force microscopy (AFM) image of a PAN nanofiber mat with 20 wt% Fe_3_O_4_. The nanofiber’s morphology shows bead-like shapes. A study by Trabesli et al. detected an agglomeration of magnetite nanoparticles in the beads using scanning electron microscopy (SEM) and energy-dispersive X-ray (EDS) spectra [208].

**Figure 7:**
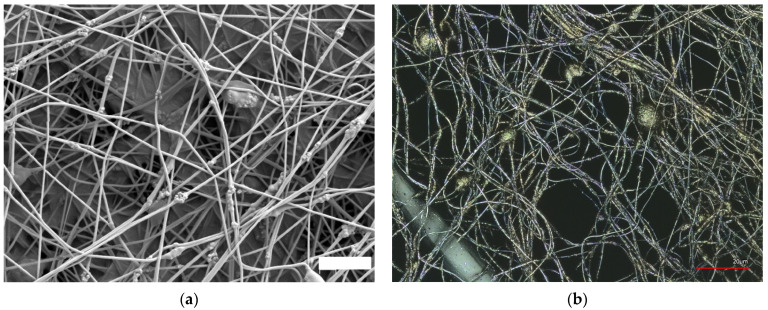
Scanning electron microscope (SEM) image showing an electrospun magnetic PAN nanofiber mat with 20 wt% Fe_3_O_4_ (**a**). Adapted from [42], originally published under a CC-BY license. Confocal laser scanning microscope image (CLSM) showing a PAN nanofiber mat with 20 wt% Fe_3_O_4_ (**b**).

Figure 7 shows scanning electron micrograph (SEM) and confocal laser scanning (CLSM) images of the electrospun magnetic PAN nanofiber mats with 20 wt% Fe_3_O_4_. As shown in Figure 6 and Figure 7, different methods, such as AFM, SEM, CLSM, or a combination of SEM and EDS, can be used to study the surface morphology of nanofiber mats [208].

Hu et al. fabricated an electrospun magnetic nanostructure of poly(caprolactone) PCL/Fe_3_O_4_ with 5 wt%, 9 wt%, 13 wt%, 17 wt%, and 21 wt% Fe_3_O_4_ nanoparticles. They applied an alternating magnetic field to generate heat and reach approx. 45 °C, which could then be transferred to cancerous tissue and cause cancer cells to die [209]. Molcan et al. prepared electrospun magnetic nanofiber mats using polyvinyl butyral and a magnetic liquid solution. These electrospun magnetic nanofibers with different magnetic nanomaterials concentrations were tested under an alternating magnetic field for magnetic hyperthermia applications and showed a temperature rise immediately after field application [210]. In addition, Song described the effect of the thickness of the magnetic nanofiber membrane made of γ-Fe_2_O_3_/polyurethane (PU) at different spinning times of 5, 10, 15, and 20 min. The maximum magnetic field strength and magnetic field frequency were 12.5 Oe and 153 kHz, respectively, to generate heat for magnetic hyperthermia, and the temperature reached was about 44.3 °C. However, the thickness of the electrospun magnetic nanofiber mats had no significant effect on heat generation [211]. Sarier et al. fabricated electrospun nanofiber mats from magnetite nanomaterials and fatty acid incorporated-poly(methacrylic acid-ethyl acrylate) for magnetic hyperthermia application. The temperature of the targeted materials was at approximately 40–48 °C [212].

In contrast, Lee et al. fabricated an electrospun magnetic nanofiber mat from the superparamagnetic nanomaterials Fe_3_O_4_ (iron oxide)/PAN (polyacrylonitrile) and used it to kill cancer cells with magnetic hyperthermia. In the experiment, they used 20 wt%, 25 wt%, and 30 wt% magnetic nanomaterials in nanofiber mats to heat the tumor cells and achieved temperatures of up to 46.8 °C, 48.8 °C, and 49.5 °C, respectively. In addition, at temperatures above 42 °C, they observed the death of cancer cells [213]. Lin et al. investigated electrospun magnetic chitosan nanofiber composites for hyperthermia treatment of tumor cells under an alternating magnetic field at a frequency of 750 kHz and a magnetic intensity of 6.4 kW [213]. According to the study by Sasikala et al., the analyzed results showed that such magnetic electrospun mats have the potential for hyperthermia treatment with endoscopic/surgical delivery and serve as a complementary postoperative tumor removal debridement treatment [214]. Matos et al. reported on electrospun magnetic nanofiber mats produced from magnetic nanomaterials with cellulose acetate membranes for magnetic hyperthermia applications [215]. Park et al. produced magnetic nanofiber mats from polyurethane and Fe_2_O_3_ using electrospinning technology and applied high-frequency magnetic fields for magnetic hyperthermia. They concluded that such electrospun magnetic nanofiber mats have the potential to be used as a substrate for magnetic materials in localized hyperthermia applications [216]. Electrospun magnetic nanofiber mats could be promising nanomaterials in magnetic hyperthermia for cancer treatment in the near future.

## 4. Drug Delivery and Electrospun Magnetic Nanofiber Mats for Cancer Treatment

The controlled release of targeted drugs is one of the most important research areas in cancer treatment, as it is one of the major challenges in medical science. Electrospinning opens new possibilities to load nanofiber mats with drugs, including thermolabile drugs, and release them in a controlled way. The excellent properties of nanofiber mats, e.g., their good mechanical stability, controlled loading and release of a variety of drugs, low toxicity, and the possibility of encapsulation, play a major role in therapy [217].

**Figure 8:**
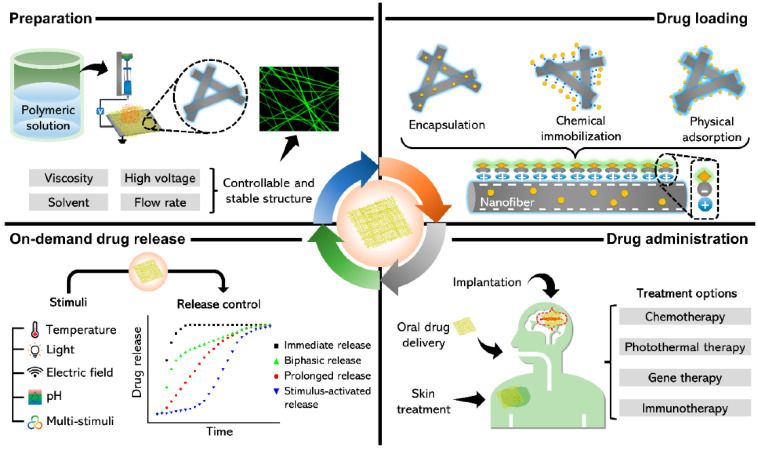
Overview showing the use of nanofiber mats in drug delivery. From [218], originally published under a CC-BY license.

Figure 8 highlights four areas of electrospinning technology for use in drug delivery. The preparation of nanofiber mats loaded with drugs is a simple process and allows for the use of a variety of different polymers. For example, depending on the electrospinning parameters, the polymers used, and other parameters, defined on-demand drug release can be realized. Drug loading and delivery can be based on encapsulation, chemical immobilization, and physical adsorption methods. The drug-loaded nanofibers can be used in many fields of therapy such as gene therapy, immunotherapy or chemotherapy, wound healing, and implants [218].

However, magnetic nanomaterial-based drugs can also be applied in cancer treatment using electrospun magnetic nanofiber mats under an alternative magnetic field due to the magnetic response of electrospun magnetic nanofiber mats and some physical and chemical properties [219,220,221,222]. This function is based on some important parameters, including the ratio of polymer to magnetic nanomaterials, the concentration of magnetic nanomaterials, and the distribution of size and magnetic nanomaterials within the electrospun magnetic nanofiber mats [223]. Due to the porous structure of electrospun magnetic nanofiber mats, the original mechanism of drug delivery is diffusion. When an external magnetic field is applied to the electrospun nanofiber mats, the magnetic nanomaterials try to align and form a barrier within the nanostructure. Small drug molecules then come out of the porous structure because the magnetic barriers limit the capacity of drug molecules with a very low diffusion rate. When the external magnetic field is removed and the alignment of the magnetic nanomaterials is damaged, the small drug molecules in the target tissue are released again. Electrospun magnetic nanofiber mats can thus also be used as a drug trigger for tumor cells when needed for cancer treatment [224,225,226,227,228,229,230,231].

**Figure 9:**
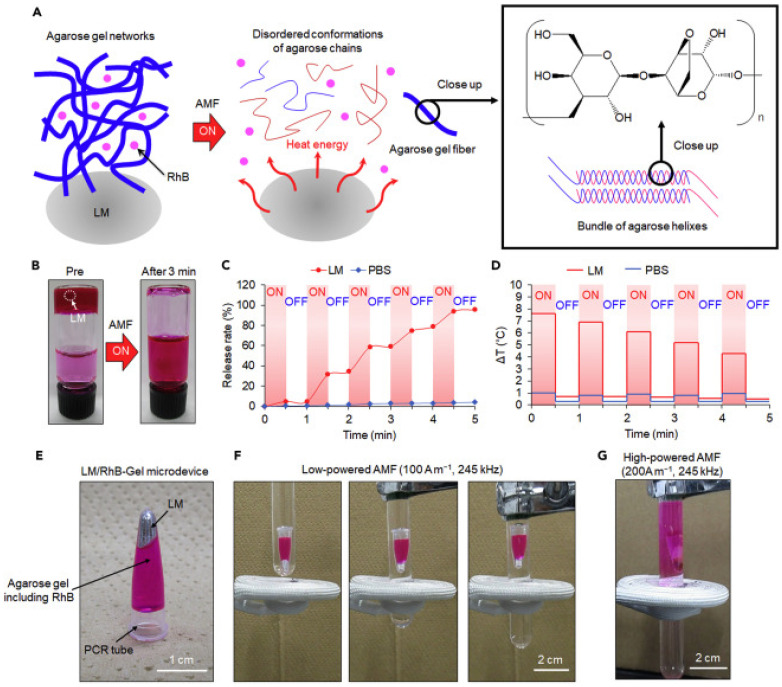
Alternating magnetic field (AMF)-controlled spatiotemporal drug release from a liquid metal (LM)–hydrogel microdevice. (**A**) Schematic illustration showing gel-sol transition of LM–agarose gel composite upon AMF induction. (**B**) A 1 mL hydrogel matrix (agarose, 2% w w^−1^; rhodamine B (RhB), 0.5 mg mL^−1^) containing LM (20 μL) in addition to 2 mL water before and after AMF (200 A m^−1^, 245 kHz) exposure for 3 min. (**C**,**D**) AMF-modulated release profile (**C**) and temperature change (**D**) of LM- or phosphate-buffered (PBS)-containing hydrogel matrix over a period of time. The “ON” stage indicates exposure to the AMF (200 A m^−1^, 245 kHz), whereas the “OFF” stage indicates the absence of AMF. (**E**) Depiction showing the LM/RhB-Gel microdevice consisting of LM (20 μL), agarose (2%, w w^−1^, 200 μL), RhB (0.5 mg mL^−1^), and a polymerase chain reaction (PCR) tube. (**F**) Sequential snapshots showing the LM/RhB-Gel microdevice electromagnetic levitation induced with low-powered AMF (100 A m^−1^, 245 kHz). (**G**) Photograph showing the LM/RhB-Gel microdevice electromagnetic levitation and RhB release behavior induced with high-powered AMF (200 A m^−1^, 245 kHz). Reproduced with permission from [226].

The study of Miyako and Yu deals with the alternating magnetic field-mediated wireless manipulations of liquid metal for use in therapeutic bioengineering. Therapeutic bioengineering applications of liquid metals (LMs) in vitro and in vivo demonstrated efficacy in magnetic cancer hyperthermia using a wireless alternating magnetic field (AMF) as well as remote manipulation of a pill-shaped microdevice based on an LM/hydrogel composite. In addition, AMFs were used effectively to eradicate tumors in vitro and in vivo with EGaIn by means of heat dissipation via eddy currents. The overview of AMF-guided spatiotemporal drug release from an LM agarose–hydrogel microdevice is shown in Figure 9 [226].

Bazzazzadeh et al. prepared poly(acrylic acid)-grafted chitosan/polyurethane/magnetic MIL-53 nanofibers with a metal–organic core–sheath for simultaneous administration of temozolomide and paclitaxel against glioblastoma cancer cells and for heat generation under an alternating magnetic field for mild hyperthermia of cells treated with magnetic MIL-53 fibers containing 5 wt% grafted-chitosan(GS)-g-poly(acrylic acid) (PAA)-paclitaxel (PTX)-temozolomide (TMZ)/polyurethane (PU) for 10 min. The study results suggest that the electrospun magnetic core–shell nanofiber mats could be used for the targeted delivery of anticancer drugs and magnetic hyperthermia applications in cancer treatment [232]. In addition, Nejad et al. described the preparation of magnetic nanofiber mats of iron oxide nanomaterials and the borate-containing anticancer drug Bortezomib (BTZ) using a catechol metal binding mechanism adapted from nature. These smart magnetic nanofiber mats show a unique conjugation of Bortezomib to their 1, 2-benzenediol (catechol) moieties for enabling pH-dependent drug delivery with simultaneous application of hyperthermia [233]. Moreover, Radmansouri et al. prepared doxorubicin hydrochloride-loaded electrospun chitosan/cobalt ferrite/titanium oxide magnetic nanofibers and investigated controlled drug release for hyperthermia tumor cell treatment. Their investigation results showed that these electrospun magnetic nanofiber mats can be used for localized cancer therapy [234]. Zhang et al. produced nanoscale materials based on the self-assembly of the mixed polymer with azo reductase-sensitive linkage (PEG-Azo-PLGA) and catechol-modified d-α-Tocopheryl polyethylene glycol 1000 succinate (TPGS) (Cat-TPGS), respectively synthesized, which provided an effective strategy for oral delivery of anti-ulcerative colitis (UC) drugs [235]. Strategies to improve the efficacy of a drug delivery system for glioma using nanoparticles were reviewed by Li et al. [236]. Sun et al. analyzed how differentiated proteins and selenium-modified chitosan induce apoptosis in HepG2 cells for the food and drug industry [237]. He et al. investigated leukemia inhibitory factor (LIF), an imperative bioactive protein that maintains stem cell self-renewal and pluripotency and is essential for mouse embryonic stem cell (mESC) culture. MgFe-layered double hydroxide (MgFe-LDH) nanoparticles can be used as a cost-effective and efficient replacement for LIF in mESC culture, according to the study [238].

## 5. Diagnostics Technology and Electrospun Magnetic Nanofiber Mats and Magnetic Nanomaterials for Cancer Treatment

The detection technique with which cancer is diagnosed plays an important role in cancer treatment. There are various kinds of detection processes in medical imaging tools and techniques including photography, microscopy, ultrasound, X-rays, computed tomography (CT) scans, magnetic particle imaging (MPI), magnetic resonance imaging (MRI), positron emission tomography (PET), etc. All of them, especially MRI and MPI, are very important tools for cancer treatment. On the one hand, MRI is very promising due to the deep penetration ability and high spatial resolution achieved using contrast agents. However, the sensitivity of T1-weighted MRI agents is low, and in T2-weighted MRI, the agents are challenging to detect in biological tissues. For this reason, magnetic nanomaterials are considered more promising T2 MRI contrast agents, and iron-based magnetic nanomaterials in particular have a longer half-life than clinically used gadolinium-based contrast agents [239,240,241,242,243]. Electrospun magnetic nanofiber mats can be loaded with a precise number of magnetic nanoparticles and implanted directly into the tumor site to enhance diagnostics. Additionally, these nanofiber mats can be used to amplify biosensor signals and improve the accuracy and sensitivity of bioassays. They are also suitable for the isolation and detection of circulating tumor cells, which is a crucial diagnostic tool. Thus, electrospun magnetic nanofiber mats are an ideal material for various diagnostic applications [244].

For example, Illés et al. used superparamagnetic nanoparticles to develop image contrast for MRI diagnosis and showed the highest values of r2 relaxivity (451 mM^−1^s^−1^) for MRI tools [245]. In addition, Khizar et al. applied magnetic cobalt ferrite nanoparticles for the combined application of MRI and magnetic hyperthermia [246]. Moreover, Islam et al. used Mn Fe_2_O_4_ nanoparticles in MRI and hyperthermia studies [247]. Nevertheless, MPI, in which magnetic nanoparticles are used, has been widely used in cancer treatment in recent times. However, the localization and concentration of magnetic nanoparticles could provide real-time 3D imaging information and can be applied in multiple medical imaging applications. Currently, electrospun magnetic nanofiber mats are also being developed for detecting tumor cells using MPI [248,249,250]. For example, Yu et al. used superparamagnetic iron oxide to study the magnetic particle imaging (MPI) tracer imaging modality and showed that the superparamagnetic iron oxide nanoparticle tracer exhibited high image contrast [247]. However, functionalized electrospun magnetic nanofiber mats and nanomaterials are new development materials for magnetic hyperthermia, which are simultaneously proving to be imaging diagnostic materials in the field of medicine [251,252,253,254,255].

## 6. Challenges and Future Research Prospective

Magnetic hyperthermia raises the temperature in cancer cells to kill them using an external alternating magnetic field generated with magnetic nanomaterials without chemical agents or severe toxicity. Although magnetic hyperthermia is the most promising cancer treatment method in modern science, there are still some limitations [256,257,258,259]. An often-overlooked factor that hinders clinical use is the inability to monitor temperature accurately, leading to incorrect temperature control [260,261,262,263,264]. In addition, among all magnetic nanomaterials, superparamagnetic iron oxide nanomaterials are the most promising materials because of their magnetic and other properties. However, these nanomaterials show high Curie temperature, which could lead to uncontrollable and irregular thermal energy, as above Curie temperature, the material shows a disordered state and is no longer thermally active due to the external field. As a result, this can damage healthy body cells [265,266,267]. In addition, magnetization decreases to zero when the external magnetic field is removed, as there are no coercive forces or remanence in the superparamagnetic nanomaterials. This helps prevent aggregation and clot formation in the bloodstream, which could cause significant undesirable effects. The United States Food and Drug Administration (FDA) has already approved several natural or synthetic polymers for the production of magnetic nanofibers for other medical applications. The majority of research studies involve only in vitro or in vivo experiments. The most significant limitation of electrospun magnetic nanofibers is that they are still in the early stages of development to detect cancer cells [268,269,270,271,272]. Nevertheless, the magnetic properties of electrospun magnetic nanofiber mats depend on the morphological structure of the nanofiber mats, such as the number and distribution of magnetic particles, aggregation or agglomeration, or the formation of beads of magnetic nanomaterials inside the nanostructure and the formation of electrospun magnetic nanofiber mats. Thus, little can be found in the literature about the magnetic properties of beads and electrospun magnetic nanofibers, the aggregation and agglomeration of magnetic nanomaterials within the electrospun magnetic nanofiber mats, and the determination of the magnetic properties. The relationship between the morphological structure, magnetic properties, and application in theranostics needs to be studied in detail for a potential outcome. The reproduction of electrospun magnetic nanofiber mats with identical morphological structures or magnetic properties is also challenging due to the agglomeration or aggregation behavior of the nanomaterials described above. In addition, these magnetic nanostructures can be implemented into the tumor without side effects, which can improve the image contrast in magnetic resonance and can also be used for continuous drug release as well as magnetic hyperthermia therapy. If medical and material scientists work together, the above challenges and limitations can be overcome using further research in this field. Electrospun magnetic nanofiber mats have shown promise for use in magnetic hyperthermia for cancer treatment. However, several challenges remain to be overcome in this area. These include the need for improved materials, better control of heating properties, and optimization of the treatment protocol. Understanding the mechanisms behind magnetic hyperthermia and its interaction with biological systems is also an important challenge.

## 7. Conclusions

The recent literature shows that significant progress has been made in the development of theranostic approaches for the treatment of cancer. Among the developed types of magnetic hyperthermia nanomaterials, electrospun magnetic nanofiber mats with superparamagnetic nanomaterials are considered one of the most effective magnetic hyperthermia materials for the near future. In addition, they exhibit excellent properties including thermal efficiency, which depends on the main function of saturation magnetization, magnetic anisotropy, intermolecular interactions, relaxation time (Néel and Brown), particle size and shape, dipolar interaction between particles, and superparamagnetic properties. These properties of the magnetic nanomaterials are very effective in generating heat under an alternating magnetic field for the targeted tumor area and in controlling targeted delivery drug triggers as well as improving the contrast quality for diagonal imaging. Furthermore, various functionalization methods and nanomaterials can be used to improve the magnetic properties of electrospun magnetic nanofiber mats or to fabricate magnetic nanomaterials for cancer theranostics, e.g., by mixing electrospinnable polymers with superparamagnetic nanomaterials or by combining the thermally conductive properties of various polymers with superparamagnetic properties and other functions added in this way. Therefore, electrospun magnetic nanofiber mats can be considered the most promising candidates for an improved cancer theranostic platform, but further research is needed. In this review, we present the magnetic nanomaterials and electrospun magnetic nanofiber mats recently developed by materials scientists in collaboration with medical researchers for magnetic hyperthermia and diagnostic nanomaterials, as well as targeted drug delivery triggers for cancer treatment, which may represent a small contribution to future research in the biomedical and pharmaceutical sector.

## Data Availability

Not applicable.
